# Bioinspired nanoparticles prevent blue-light-induced skin hyperpigmentation via FZD2-TYR-melanin pathway

**DOI:** 10.1016/j.mtbio.2025.102288

**Published:** 2025-09-04

**Authors:** Xiaoqi Chen, Tong Wu, Zijun Chen, Jia Zhang, Yuqi Zhou, Qi Wang, Bo Wang, Zeqian Wang, Xiaodong Jin, Shishi Xiong, Tong Zhang, Shanshan Gao, Jingjing Ma, Ziwei Deng, Xutao Chen, Chunying Li, Zhe Jian

**Affiliations:** a Department of Dermatology, Xijing Hospital, Fourth Military Medical University, Xi'an, 710032, Shaanxi, PR China; b Faculty of Dentistry, The University of Hong Kong, Hong Kong, 999077, PR China; c Department of Immunology, School of Basic Medicine, Fourth Military Medical University, Xi'an, 710032, Shaanxi, PR China; d Key Laboratory of Applied Surface and Colloid Chemistry, Ministry of Education, School of Materials Science and Engineering, Shaanxi Normal University, Xi'an, 710119, PR China

**Keywords:** Polydopamine nanoparticles, Cuttlefish ink nanoparticles, Blue light, Melanin, FZD2

## Abstract

Melanin is the natural physiological defense for skin, but excessive melanin production can result in conditions like hyperpigmentation and melasma. In addition to UV light, visible light, especially blue light, has been recognized as an extra factor contributing to cutaneous hyperpigmentation and premature photoaging. However, there are currently no effective protective agents against blue light. Within this research, we obtained polydopamine nanoparticles (PDA NPs) and cuttlefish ink nanoparticles (CINPs), which are inspired by endogenous melanin. Our research confirmed that PDA NPs and CINPs shared similar structural and functional properties with natural melanin. Otherwise, these nanoparticles not only exhibited excellent photostability and broad-spectrum ultraviolet–visible light absorption capabilities but also possessed superb biocompatibility and antioxidant properties. Experiments conducted in vitro and in vivo verified that PDA NPs and CINPs could effectively hold back melanin production and alleviate pigmentation. Furthermore, we found that the underlying mechanism by which PDA NPs and CINPs reduced melanin formation was through inhibition of the FZD2-TYR-melanin signaling pathway. Taken together, our findings not only demonstrate that PDA NPs and CINPs are powerful anti-blue light agents but also provide an in-depth mechanism of these nanoparticles in the inhibition of pigment formation.

## Graphical abstract

**Figure:**
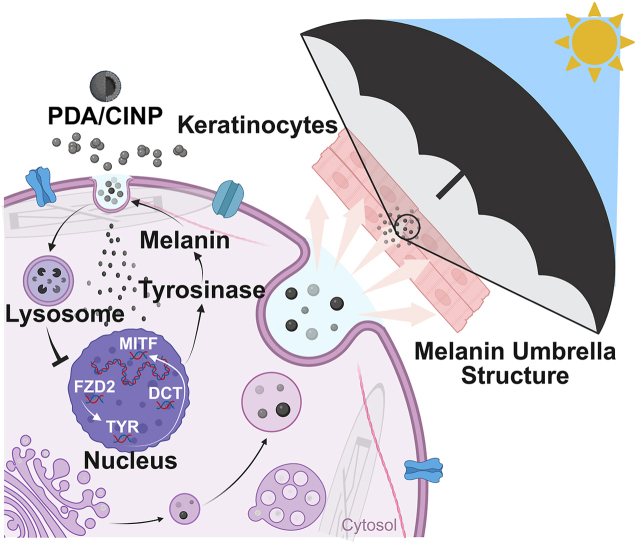
Bioinspired nanoparticles, following lysosomal metabolism within keratinocytes, effectively suppress FZD2-mediated melanogenesis, thereby preventing skin hyperpigmentation induced by blue light exposure. This mechanism relies on the targeted degradation of the nanoparticles within lysosomes, leading to the inhibition of the FZD2 signaling pathway—a key regulator of melanin production. As a result, the formulation offers a novel therapeutic strategy for mitigating pigmentation disorders caused by blue light, highlighting its potential for application in dermatological and cosmetic treatments.

## 1 Introduction

Solar radiation is the main determinant of hyperpigmentation [1,2]. However, in recent years, increasing research has found that hyperpigmentation induced by visible light is more persistent than UV-induced pigmentation [[3], [4], [5]] owing to its capacity to penetrate the epidermis and access the dermis, as well as its prolonged stimulation of melanocytes. Blue light, which falls within the wavelength range of 400–500 nm, is categorized as high-energy visible (HEV) light and has been recognized as an auxiliary contributor to skin pigmentation. Excessive blue light exposure has been demonstrated to disrupt the skin's circadian rhythm [6,7], induce inflammation, generate excessive reactive oxygen species (ROS) [8,9], lead to collagen degradation, and accelerate photoaging [10]. The prevention of blue light-induced pigmentation represents a significant area of research interest.

Currently, the risk of blue light-induced hyperpigmentation can be mitigated primarily through the use of sunscreens containing antioxidants [11,12], such as vitamin C [13,14], and physical protective ingredients [[15], [16], [17], [18], [19]], including zinc oxide and titanium dioxide. Despite the validated effectiveness of these ingredients in counteracting blue light-induced skin damage, significant drawbacks persist. For example, vitamin C is highly susceptible to light and air, and is readily oxidized, resulting in a notable reduction in its efficacy. The reflective and scattering properties of zinc oxide and titanium dioxide against UV light are well-established, but these materials cannot block all wavelengths of blue light completely. Consequently, their protective effect may be diminished, particularly in cases of prolonged exposure to high-energy visible (HEV) light. Therefore, although certain ingredients, including vitamin C, zinc oxide, and titanium dioxide, have been used in protecting against blue light, each has its inherent limitations. It makes sense to develop novel and effective sunscreens that not only protect against UV rays but also against damage caused by blue light.

Melanin is a natural pigment that absorbs blue light and possesses antioxidant properties that facilitate the neutralization of free radicals within the body [20], thereby safeguarding cells from the detrimental effects of oxidative damage [21]. Melanocytes gradually produce melanin in response to exposure to blue light, which is then transferred to keratinocytes for protection [22]. However, this process is often insufficiently rapid to meet optimal protective requirements. Inspired by the natural melanin in the skin, we introduced bioinspired melanin-like substances, polydopamine nanoparticles (PDA NPs), and cuttlefish ink nanoparticles (CINPs), to supplement the insufficient melanin production by melanocytes. The physicochemical properties of PDA NPs and CINPs resemble those of natural melanin in melanocytes, effectively preventing the activation of melanocytes by blue light. This meets the cosmetic demand for skin whitening while providing critical protection against photoaging and even carcinogenic skin lesions.

PDA is a synthetic black polymer derived from dopamine (DA) and forms spherical nanoparticles measuring 100–200 nm in size. It has been demonstrated that PDA-based formulations serve as effective sunscreen alternatives for preventing UV-mediated skin photoaging, while concurrently potentiating antioxidative and anti-inflammatory responses [23,24]. Studies have shown that PDA can be used as a topical sunscreen-like formulation to protect against UV-induced photoaging, with enhanced antioxidant and anti-inflammatory properties [25,26]. PDA has a broad-spectrum absorption band [27], making it a suitable bioinspired melanin that can absorb not only ultraviolet light but also blue light. CINPs are natural melanin nanoparticles extracted from the ink sacs of cuttlefish. Their structure mainly consists of indole-5,6-quinone (DHI) and 2-carboxy-indole-5,6-quinone (DHICA), and they have various types of functional groups (e.g., amino, hydroxyl, and carboxyl groups) as well as an abundance of metal chelating sites. The particles, with diameters ranging from 100 to 200 nm, provide a similar photoprotection to human melanin [[28], [29], [30]]. CINPs also exhibit good biocompatibility and a wide range of biological activities [31], including anti-inflammatory [32], antioxidant [33], metal ion chelation, anti-tumor, and antibacterial effects [30,34,35], which suggest a significant potential for the prevention of pigmentation and photoaging.

Herein, we presented a comprehensive investigation into the photoprotective capacities of synthetic PDA NPs and natural CINPs against blue light exposure in vitro and in vivo. Then, we further explored the in-depth molecular mechanism by which PDA NPs and CINPs prevented blue-light-induced hyperpigmentation. Our study demonstrated that PDA NPs and CINPs not only absorbed and scattered blue light by supplementing exogenous melanin but also regulated endogenous melanin synthesis by influencing the FZD2-TYR-melanin pathway.

## 2 Materials and methods

### 2.1 PDA NPs preparation

PDA NPs were synthesized using oxidative self-polymerization by adding 2 g/L of dopamine hydrochloride (Rishi Bio, China) to a 30 % ethanol solution for mixing and dissolution, followed by mixing with 0.1 mol/L of ammonia. Reaction lasts 24 h at 37 °C. The material was then spatialized at 8000 rpm for 30 min, washed several times with ddH_2_O, dried, and frozen overnight to obtain the lyophilized powder of the samples.

### 2.2 Purification of cuttlefish ink melanin

Cuttlefish (∼800 g) was obtained from Sincere Aquatic Products Co. Melanin was extracted from cuttlefish ink sacs using a combination of wash centrifugation and enzymatic hydrolysis. The ink from the sac was mixed with double-distilled water and subjected to repeated centrifugation (10,000 rpm, 15 min) repeatedly for 6–8 cycles. The resulting precipitate was then subjected to hydrolysis with alkaline protease. Subsequently, the mixture was heated to inactivate the protease, after which centrifugation, washing, and collection of the precipitate were performed. This constituted the melanin nanoparticles.

### 2.3 Characterization of PDA NPs and cuttlefish ink melanin

The morphology and structure of PDA NPs and CINPs were observed by transmission electron microscopy of magnified samples at 200 kV (TEM, JEOL, JEM-F200, Japan). A NanoCoulter counter (Ruixing Technology Co., Ltd., Shenzhen, China) was used in the particle-by-particle analyses to evaluate the size distribution and zeta potential, and a nano-well chip with a measurement range of 60–200 nm was selected for the experiments. Measurement of UV–visible (UV–Vis) absorption curves of samples using a UV–Vis photometer (PerkinElmer, Lambda 650, USA). Fourier Transform Infrared (FT-IR) Specification with Thermo Fisher NICOLET IS50 Instrument.

### 2.4 Extraction of primary human melanocytes and keratinocytes

The specimens were obtained from the foreskin tissue of patients aged 8–30 years undergoing circumcision. The foreskin was cut open and rinsed, then immersed in 75 % ethanol for 50 s to remove subcutaneous fat tissue. After soaking in a medium containing 10 % penicillin-streptomycin for 5 min, the tissue was split into 1 cm pieces and soaked in 0.25 % collagenase I (Gibco 17018029) overnight at 4 °C. The epidermis was torn off to separate it from the tissue, cut into small pieces, digested with trypsin for 7 min, and then centrifuged. The cells were cultured in specific media for melanocytes (Gibco M254500) and keratinocytes (Gibco) at 37 °C and 5 % CO_2_. One week later, melanocytes and keratinocytes were separated and purified by tryptic digestion. The study followed the guidelines of the Declaration of Helsinki and was approved by the Medical Ethics Committee of the First Affiliated Hospital of Fourth Military Medical University (license number: KY20233160-1). Receive written informed consent from all minor and adult circumcision donors.

### 2.5 Coculture of primary human melanocytes and human keratinocytes

Inoculation of purified melanocytes into 6-well plates, while extracted keratinocytes were inoculated into Transwell chambers. The ratio of melanocytes to keratinocytes was 1:3. Once the cells had reached 80 % density, the Transwell chambers were placed in the wells for co-culture, which lasted 24–48 h. All cell experiments were repeated 6 times.

### 2.6 Observation and measurement of intracellular reactive oxygen species (ROS)

Intracellular ROS were evaluated using 2,7-dichlorodihydrofluorescein diacetate (DCFH-DA). Cells were bound to PDA NP and CINP for 6 h and then exposed to blue light (60 J/cm^2^) for 24 h. Subsequently, keratinocytes (KC) and melanocytes (MC) on a dark background were dyed with DCFH-DA for 30 min and the fluorescence strength was detected by confocal microscopy (Zeiss LSM880).

### 2.7 Cell viability assay

Biocompatibility of PDA NPs and CINPs were assessed by cell viability assay (CCK-8, Glpbio, CA, USA). Keratinocytes were separately incubated with various concentrations of PDA nanoparticles and CINPs over 24 h. The cultured cells were then washed three times with PBS, and 100 μL/well of CCK-8 working buffer was added to the cells (dilute the working solution at a ratio of 1:10). After 2 h of incubation, the absorbance (OD) of enzyme labeling was recorded at 450 nm (ThermoScientific, USA).

### 2.8 Enzyme-linked immunosorbent assay (ELISA)

The ELISA kit detects endothelin-1 (ET-1) expression levels in KC cell supernatants (E-EL-H0064; Elabscience; China), and all procedures are carried out following the guidelines of the manufacturer.

### 2.9 Preparation of sunscreen

PDA NPs and CINPs were incorporated into sunscreen formulations at a concentration of 5 wt%, which was achieved by adding 2.5 g of nanoparticles to 50 g of emulsion matrix.

### 2.10 Animal models

Female brown guinea pigs (4–6 weeks old) were purchased from Shaanxi Junxing Biotechnology Co., Ltd. and kept in a pathogen-free environment designated by the Animal Centre of Fourth Military Medical University. The guinea pigs were randomly divided into 2 groups after 1 week of adaptation to the environment, and the control group was shaved and depilated only. The dorsal skin of guinea pigs in the experimental group was divided into four sections, corresponding to four treatments: application of emulsions containing 5 wt% PDA NPs and blue light irradiation; application of emulsions containing 5 wt% CINP and blue light exposure; direct application of emulsions and blue light exposure; and blue light exposure. In this case, 5 wt% of PDA NPs and CINPs were mixed with emulsion and used as nanomaterial carriers, respectively, for topical applications only; the group of directly applied emulsions and blue-light irradiation was intended to exclude the protective influence of the emulsions from blue light. All animal sample experiments were repeated 4 times. All procedures were performed following the ethical guidelines approved by the Fourth Medical University Subcommittee on Research Animal Care (approval number: IACUC-20241484).

### 2.11 Blue light source and irradiation protocol

The blue light source consisted of an LED matrix embedded in 4 × 4 cm and 11 × 14 cm circuit boards (Xuzhou Ai Jia Electronic Technology Co., Ltd.). The light source at 400–430 nm is used for individual irradiation of animal and cell experiments with a power output of 300 mW/cm^2^. The irradiance was monitored using a blue light spectrometer (OHSP350B, Hangzhou Hongpu Technology Co., Ltd.), ensuring an accumulated dose of 60 J/cm^2^ per session. Guinea pig skin was divided into four 2 × 1 cm regions, corresponding to different treatments, and irradiated with blue light three times per week for three weeks.

### 2.12 Staining with hematoxylin and eosin

Skin tissue sections were formalin-fixed, paraffin, and cut into 5 μm pieces. The sections were stained after the removal of paraffin and finally blotted using hematoxylin and eosin (H&E stain) and examined histologically under a light microscope.

### 2.13 Fontana Masson staining

Paraffin sections were deparaffinized, and skin tissue was incubated in Fontana ammoniacal silver solution at 56 °C for 40 min. Rinse 5–6 times with distilled water, then rinse with 5 % sodium thiosulfate to remove areas of non-melanin staining. Nuclear staining was performed with neutral red, after which the sections were dehydrated and mounted in resin for microscopic observation (Regenbio DJ0021).

### 2.14 Measurement of melanin content

Remove the top cavity of the cell culture system and suction out the medium. Add PBS to the lower chamber, followed by cell lysis with 250 μL of a PMSF-containing cell lysis buffer. The cells were scraped, and the cell suspension was collected in suitable centrifuge tubes. The solution was concentrated by centrifugation at 12,000 rpm for 15 min and the melanin precipitate was collected at 4 °C. After transferring the supernatant, the precipitate was dissolved in 330 μL of 10 % NaOH containing DMSO, vortexed to mix, and inspected at 85 °C for 30 min. The mixture was added to a 96-well plate (100 μL each) and the absolute absorbance was calculated at 405 nm. The melanin content was measured by the formula (OD test group - OD blank group)/(OD control group - OD blank group).

### 2.15 Tyrosinase assay

Cells are lysed with 1 % Triton X-100, left at −80 °C for 30 min, and then thawed at 37 °C. The solution is centrifuged at 1000 g for 10 min. The supernatant was transferred to a 96-well plate, 10 μL of levodopa mixture (2 mg/mL) was added to each well, and incubated at 37 °C for 20 min, then the absorbance was measured at 475 nm using an enzyme labeling. The tyrosinase activity was calculated as follows: (OD test group - OD blank group)/(OD control group - OD blank group).

### 2.16 Immunofluorescence assay

Paraffin-embedded tissue sections were deparaffinized and antigenically repaired with sodium citrate buffer (10 mM, pH 6.0). After blocking with goat serum, antibodies were added and incubated at 4 °C overnight. The following antibodies were used: FZD2 (24272-1-AP, Proteintech, 1:100), Wnt6 (24201-1-AP, Proteintech, 1:100), Melanin A (#ab187369, Abcam, 1:100) and Lysosome (L7528, ThermoFisher, 1:1000). Subsequently, fluorescent antibodies, i.e., Cy3 (Zhuangzhi Bio, Xi'an, China) and FITC (Zhuangzhi Bio, Xi'an, China) were used. Stain the nucleus with Hoechst 33,258 (Beyotime, Shanghai, China). Immunofluorescence images were acquired using a confocal laser microscope (Zeiss LSM880).

### 2.17 Western blotting

Protein expression levels in tissues and melanocytes were detected by Western blotting. After treatment, the extracted cells were rinsed three times with PBS and the proteins were lysed with RIPA protein buffer containing PMSF for 25 min. Protein levels were determined by the BCA method (Elabscience, Wuhan, China). SDS-PAGE was performed to separate the protein samples, which were then transferred to a PVDF membrane. Incubate membrane with the antibody at 4 °C overnight: GADPH (ab8245, Abcam, 1:5000), FZD2 (24272-1-AP, Proteintech, 1:1000), Wnt6 (24201-1-AP, Proteintech, 1:1000). Chemiluminescence was performed using a Bio-Rad imaging system. Band densities were analyzed using ImageJ and relative protein expression was normalized to GADPH.

### 2.18 Quantitative real-time PCR (RT-qPCR)

Total RNA was purified with Trizol(Xi'an Chemical Reagent Factory) and then reverse transcribed into cDNA (Invitrogen). Finally, RT-qPCR (RR036A, TaKaRa, Tokyo, Japan) was performed. Primers were synthesized by Zingke Biotechnology (Beijing, China).RT-qPCR was performed at 95 °C for 3 min, followed by 40 cycles: 95 °C for 15 s, 60 °C for 15 s, 72 °C for 15 s, and finally melt curve analysis. Relative gene expression was quantified using the 2^(−ΔΔCT) method.

### 2.19 Animal sample collection and RNA preparation

Quantification and integrity assessment of RNA in animal skin samples was performed using a Bioanalyzer 2100 system (Agilent Technologies, California, USA) and an RNA Nano 6000 analysis kit. Total RNA was used for library preparation and sequencing. Purified mRNA from total RNA was obtained from poly-T oligonucleotide-conjugated beads. RNA was broken using divalent cations at high temperatures. First-strand cDNA was synthesized using primers for random hexamers and M-MuLV reverse transcriptase, followed by second-strand synthesis. After adenylation of the 3′ end, a ligand protein with a hairpin loop was ligated and used for hybridization. The cDNA fragments (370–420 bp) were purified using an AMPure XP system (Beckman Coulter, USA) and amplified by PCR using Phusion high-fidelity DNA polymerase, universal primers, and exponential primers. PCR products were purified and evaluated on an Agilent Bioanalyzer 2100 system. Cluster analysis was performed in the cBot Cluster Generation System (TruSeg PE Cluster Kit v3-cBot-HS, Illumina) followed by sequencing on the Illumina Novaseq platform using 150 bp double-ended read lengths. Pure data was obtained by removing low-quality reads and adapter contamination from the raw data, and quality metrics such as Q20/Q30 and GC levels were calculated. Downstream final analyses were performed using high-quality, pure data.

### 2.20 Transcriptomic sequencing analysis

To detect mRNA expression by RT-qPCR, total RNA was purified with Trizol (Xi'an Chemical Reagent Factory), and then reverse-transcribed into cDNA by Superscript First-Strand Synthesis Kit (Invitrogen), followed by RT-qPCR with PrimeScript RT Master Mix (Cat# RR036A, TaKaRa, Tokyo, Japan). Primers were synthesized using TsingKe Biotechnology (Beijing, China). RT-qPCR was performed at 95 °C for 3 min, followed by 40 cycling cycles: 95 °C for 15 s, 60 °C for 15 s, 72 °C for 15 s, and finally melting curve analysis. Curve analysis. Quantification of relative gene expression was performed using the 2^ (-ΔΔCT) method.

### 2.21 Statistical analysis

The results of each experiment are represented by mean ± standard deviation (SD). The significance of the experimental results data was calculated by Student's unpaired *t*-test and one-way analysis of variance (ANOVA). Results were regarded as significant when p was less than 0.05.

## 3 Results

### 3.1 Characterization of PDA NPs and CINPs

**Fig. 1:**
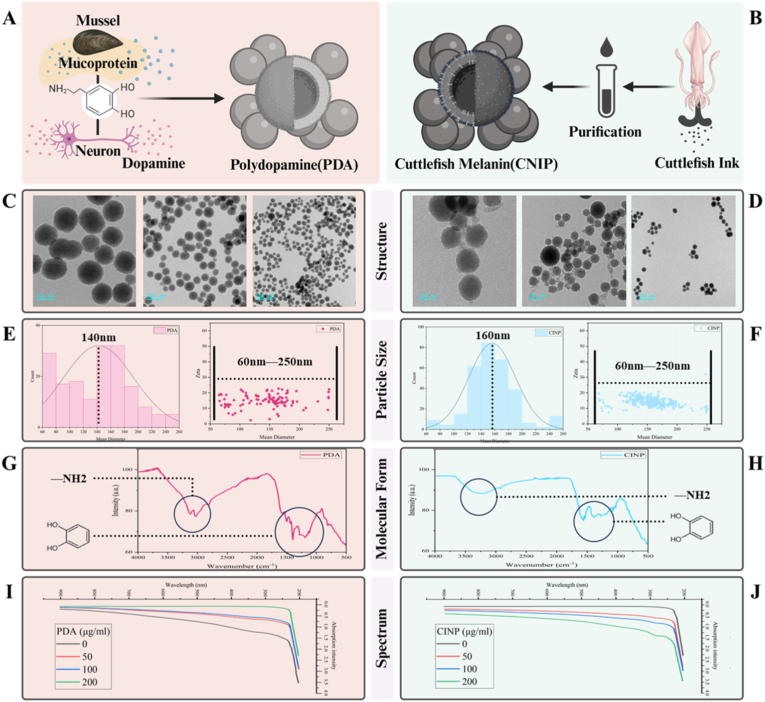
**Characterization of PDA NPs and CINPs.** (A, B) The flow chart presents the synthesis of PDA NPs and CNIPs. (C, D) TEM images of PDA NPs and CNIPs. Scale bar = 500 nm. (E, F) Quantitative analysis of particle size distribution of PDA NPs and CINPs (n = 100). (G, H) FTIR spectra of the PDA NPs、CINPs. (I, J) UV–Vis absorption spectra of PDA NPs, CINPs, and PBS solutions at different concentrations (50, 100, 200 μg/mL).

PDA NPs were synthesized at room temperature through the oxidation and self-polymerization of dopamine hydrochloride in an alkaline water-ethanol solution, and CINPs were obtained using a combination of wash centrifugation and enzymatic hydrolysis (Fig. 1A and B). TEM was employed to characterize the morphological and structural features of both PDA NPs and CINPs (Fig. 1C and D). PDA NPs were observed to display a monodisperse spherical architecture, measuring 142.3 nm in mean diameter, and the surface zeta potential was determined to be −14.93 mV. CINPs exhibited a broad size distribution (mean diameter: 168 nm) and varied geometries, concomitant with a zeta potential measurement of −15.00 mV (Fig. 1E and F). There were no notable alterations in the PDA NPs or CINPs over the two-week observation period. This indicates that both types of particles possess stability and resistance to degradation. The FT-IR analysis revealed an intense and broad absorption band around 3100 cm^−1^ corresponding to the stretching vibration of amines. The stretching vibration of catechol was identified within the range of 1350–1600 cm^−1^ (Fig. 1G and H). These findings confirm that the synthesized materials are indeed polydopamine and cuttlefish ink melanin [36,37].

The UV–Vis absorption spectra of both materials were examined at various concentrations (50, 100, 200 μg/mL) across wavelengths of 200–800 nm (Fig. 1I and J). The absorption gradually decreased from 200 nm, with CINPs showing a significant absorption peak around 300 nm, while no prominent peaks were observed for PDA NPs. At a concentration of 50 μg/mL, both PDA NPs and CINPs exhibited strong absorption, indicating their capacity to absorb and scatter blue light effectively. It can thus be concluded that there is a similarity in the morphologies and functions of both CINPs and PDA NPs, which are characterized by their stable UV–Vis absorption spectra and structural stability [38,39].

### 3.2 PDA NPs and CINPs mitigate blue light-induced skin pigmentation in animal models

**Fig. 2:**
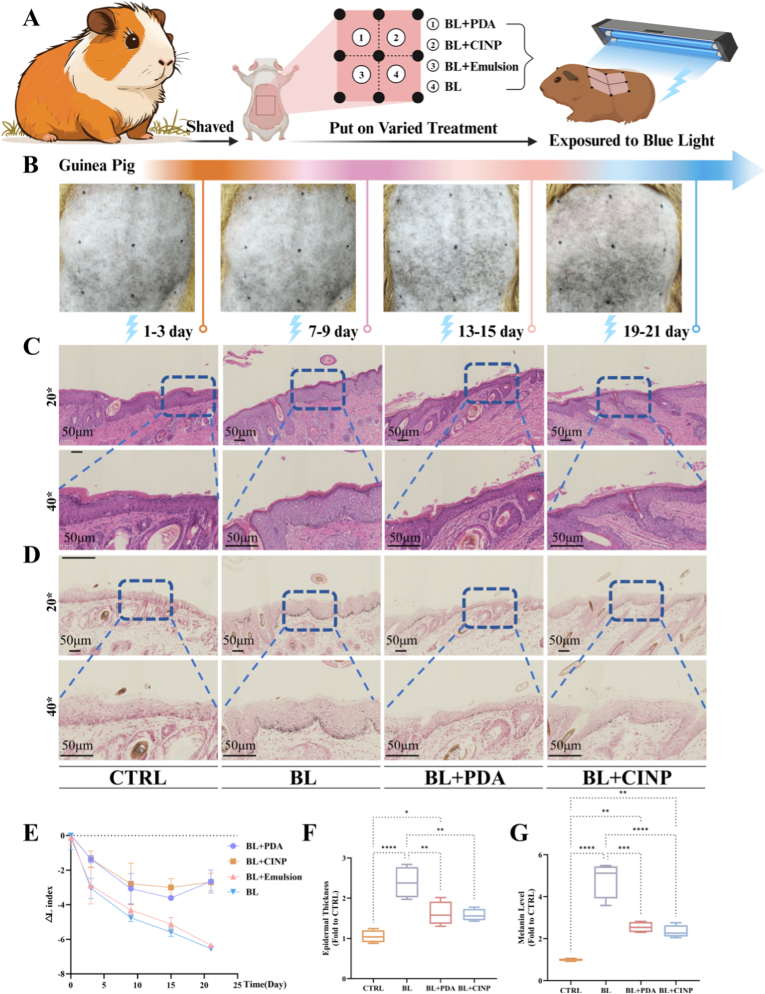
**PDA NPs and CINPs alleviate blue light-induced pigmentation in guinea pig skin.** (A) The schematic diagram shows the topical application of nano melanin on the dorsal skin of a brown guinea pig. BL + PDA: topical application of emulsion containing PDA followed by blue light irradiation; BL + CINP: topical application of emulsion containing CINP followed by blue light irradiation; BL + Emulsion: topical application of emulsion alone followed by blue light irradiation, in this group, to exclude that the topical application of emulsion affects the results; BL: blue light only. Exposure to blue light for 21 days. (B) Representative images of dorsal hyperpigmentation in brown guinea pigs at different time points after treatment. (C) H&E staining images of brown guinea pig skin. (D) Fontana-Masson staining of melanin in the skin of brown guinea pigs. (E) Changes in skin pigmentation were assessed spectrophotometrically and ΔL values were quantified at different time points. (F) Quantitative analysis of epidermal thickness in C and melanin level in D. All data are presented as SD ± mean. ∗*P* < 0.05, ∗∗*P* < 0.01, and ∗∗∗*P* < 0.001. (For interpretation of the references to colour in this figure legend, the reader is referred to the Web version of this article.)

The cutaneous tissue of brown guinea pigs, which exhibits high structural homology to human skin, harbors melanocytes within the epidermal basal layer, thus enabling the induction of pigmented phenotypes [40,41]. The guinea pig's dorsal skin was divided into four 2 × 1 cm sections corresponding to four treatments: application of an emulsion containing 5 % wt PDA or CINP (PDA NPs and CINPs were prepared by mixing with the emulsion); direct application of the emulsion and then exposure to blue light; direct exposure to blue light (Fig. 2A). Each region was exposed to blue light three times per week at a dose of 60 J/cm^2^ per exposure. After 21 days of irradiation, the areas treated with blue light exposure and the emulsion showed pronounced pigmentation, while pigmentation was significantly reduced in the areas treated with PDA and CINP nanoparticles (Fig. 2B). The ΔL values indicate the darkness level of the skin, reflecting increased pigmentation. To ascertain the ΔL values, weekly spectrophotometric measurements of skin pigmentation were conducted, comparing the L values from each week to the baseline. The results confirmed that the ΔL values were consistent with visual observations (Fig. 2E). We also administered emulsions containing varying concentrations (1 %, 2.5 %, 5 %, and 10 % wt) of PDA NPs or CINPs via topical application to the dorsal skin of guinea pigs, followed by blue light irradiation, in order to determine the minimum effective concentration. The results indicated that 5 % wt was the minimum effective concentration, as illustrated in Fig. S1.

Histological analysis of skin biopsies stained with hematoxylin and eosin showed that the epidermal layer demonstrated marked thickening in the blue light-exposed group relative to controls, while no significant epidermal hyperplasia was detected in the nanoparticle-treated samples (Fig. 2C). The Fontana Masson staining further confirmed the increased melanin deposition in the basal layer of the blue light-exposed group, and a significant decrease in the nanoparticle-treated group's melanin levels were significantly reduced (Fig. 4D). Quantitative analysis was performed on epidermal thickness based on H&E staining and melanin content based on Fontana Masson staining (Fig. 2F). Overall, the results of our animal model experiments indicate that sunscreens containing PDA NPs and CINPs effectively protect the skin from blue light-induced pigmentation by supplementing exogenous melanin.

In order to verify the blue light protection advantages and biosafety of PDA NPs/CINPs, a positive control (iron oxide3, Fe_2_O_3_) was added in this study for a parallel comparison with a conventional sunscreen (zinc oxide4, ZnO). A mixture of 5 % wt PDA NPs, CINPs, ZnO or Fe_2_O_3_ emulsion was topically applied to the backs of guinea pigs and tested after blue light irradiation. Skin pigmentation (ΔL value), histological changes (H&E staining: epidermal thickness, Fontana-Masson staining: melanin deposition) were detected and the results are shown in Fig. S2. Significant pigmentation was observed in the ZnO group. Whereas, no significant pigmentation was observed in the PDA NPs, CINPs and Fe_2_O_3_ groups. Histological analysis consistently showed that epidermal thickness and melanin deposition were significantly higher in the ZnO group than in the NPs and Fe_2_O_3_ groups. Biocompatibility experiments confirmed that Fe_2_O_3_ showed significant cytotoxicity at >120 μg/mL, and ZnO showed dose-dependent toxicity in the range of 2–7 μg/mL. Our results show that PDA NP and CINPs combine excellent blue light protection (equivalent to Fe_2_O_3_) and higher biological safety (significantly superior to ZnO/Fe_2_O_3_).

### 3.3 Blue light induces the proliferation of keratinocytes and melanogenesis

**Fig. 3:**
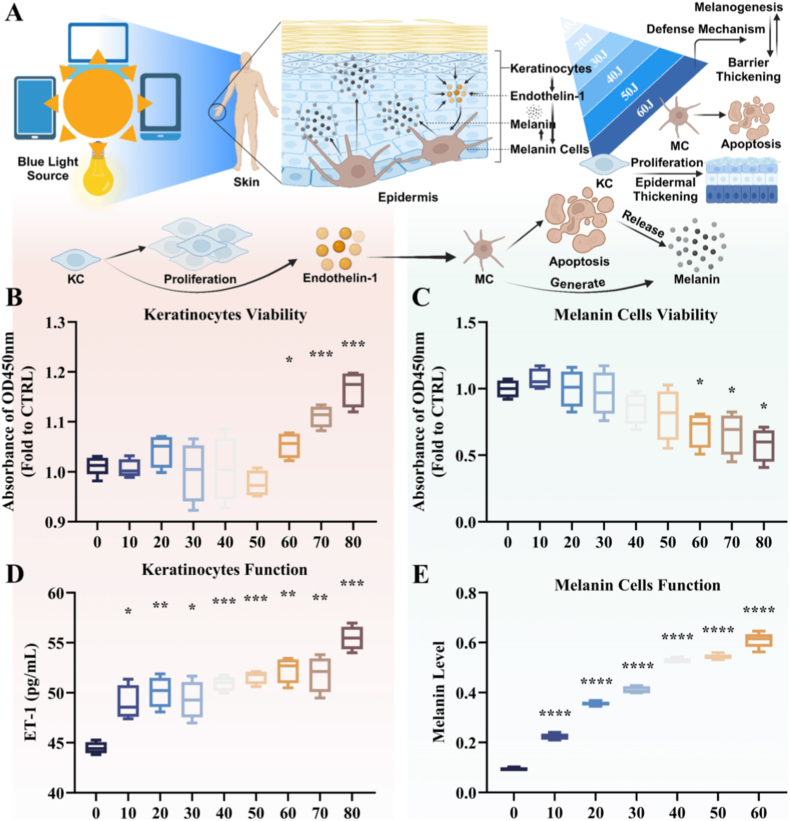
**Blue light induces proliferation and melanogenesis in epidermal cells.** (A) Schematic diagram of blue light damage to epidermal cells: Blue light can stimulate the proliferation of keratinocytes, while the viability of melanocytes decreases with increasing dosage. Blue light also promotes keratinocytes to secrete endothelin-1, which in turn stimulates melanocytes to produce significant amounts of melanin. Blue light-induced skin changes involve defense mechanisms such as stratum corneum thickening and melanin production. (B) The impact of varying doses of blue light on the viability of human primary keratinocytes (KCs) was assessed using the CCK8 method. (C) The effect of varying doses of blue light on the viability of human primary melanocytes (MCs) was also evaluated using the CCK8 method. (D) The secretion levels of the ET-1 cytokine from KC cells exposed to varying doses of blue light were measured using the ELISA method. (E) Irradiating MC cells with different doses of blue light, the melanin content in the cells was determined using the NaOH method. All data are demonstrated as SD ± mean. ∗*P* < 0.05, ∗∗*P* < 0.01 and ∗∗∗*P* < 0.001. (For interpretation of the references to colour in this figure legend, the reader is referred to the Web version of this article.)

Similar to other studies, varying doses of blue light have different impacts on human primary keratinocytes (KCs) and melanocytes (MCs) [42]. Using the CCK8 assay, the results returned that KC showed a significant increase in proliferation under a blue light dose of 60 J/cm^2^ (Fig. 3B), while MCs viability began to decline at the same dose (Fig. 3C). Additionally, the secretion of the endothelin-1(ET-1) from KCs increased progressively with higher blue light doses (Fig. 3D), and MC melanin production also rose in response to increasing blue light exposure (Fig. 3E). At higher doses of blue light, MCs apoptosis was observed. However, the melanin content in these cells did not show a substantial decrease, suggesting that excessive doses of blue light induce apoptosis in MCs, leading to the release of melanin granules as the cells rupture.

**Fig. 4:**
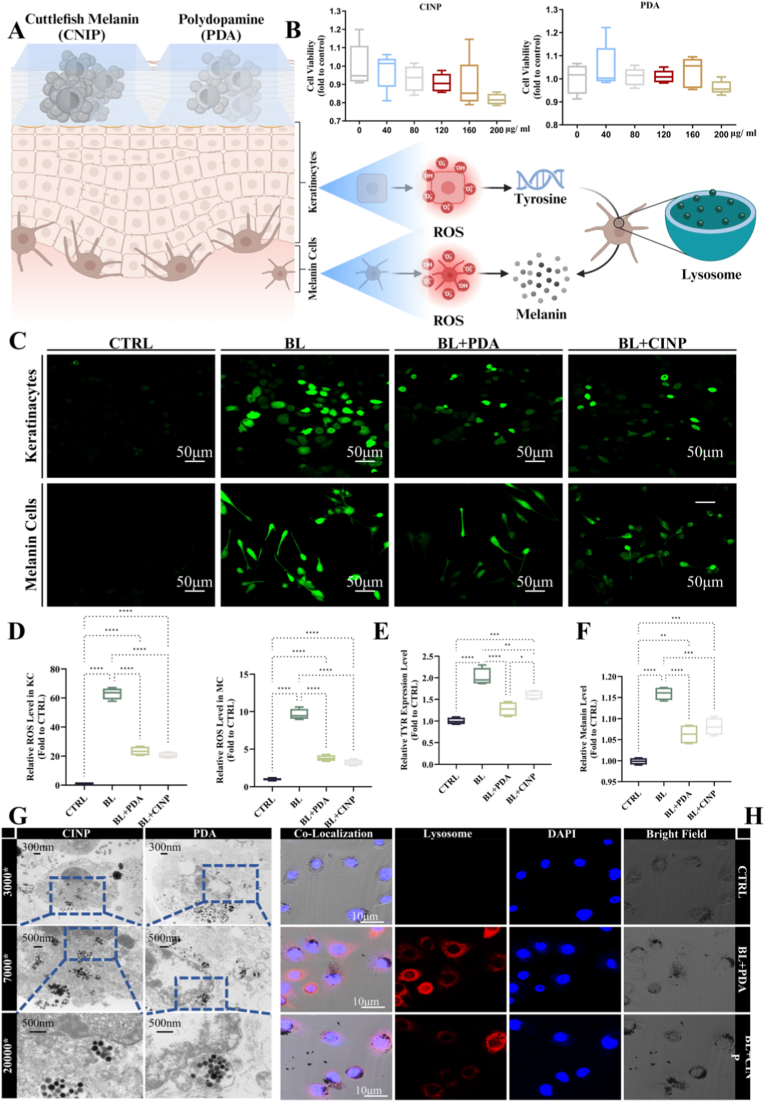
**PDA NPs and CINPs inhibit blue light-induced oxidative stress and melanin production in epidermal cells.** (A) Schematic representation of PDA NPs, CINPs reducing blue light-induced ROS production, elevated tyrosinase expression, and increased melanin content. (B) The toxic responses of different concentrations of (0, 40, 80, 120, 160, 200 μg/mL) PDA NPs, and CINPs in KC cells were detected using the CCK8 method. (C) Representative fluorescence pictures and ROS level quantification in KCs and MCs intervened with 40 μg/mL PDA NPs, and CINPs after blue light irradiation (60 J/cm^2^) for 24 h. DCFH-DA (green) was applied to quantify ROS levels, scale bar = 50 μm. (D) The ROS levels in KC and MC cells after different treatments were counted and statistically analyzed. (E) In the KC-MC cell co-culture system, the mRNA expression of TYR in MC cells of control, blue light, blue light + PDA NPs, and blue light + CINPs groups was detected using RT-qPCR. (F) Changes in melanin content in MC cells in control, blue light, blue light + PDA NPs, and blue light + CINPs groups were detected using the NaOH method. (G) The uptake of PDA NPs and CINPs by KC cells and their distribution within KC cells were visualized by cellular transmission electron microscopy (TEM), where the nanoparticles formed a vesicle-encapsulated structure within the cytoplasm, aggregated around the nuclear membrane, and were exocytosed and metabolized by cytosolic action, Scale bar = 500 nm. (H) Laser Scanning Confocal Microscopy (LSCM) was used to picture lysosomes after blue light irradiation of PDA NPs, and CINPs. Lysosomes were in red (LysoTracker Red and −99); Nuclei were in blue (Hoechst 33,258); PDA NPs and CINPs in KC cells were in black, Scale bar = 10 μm. All data are demonstrated as SD ± mean. ∗*P* < 0.05, ∗∗*P* < 0.01 and ∗∗∗*P* < 0.001. (For interpretation of the references to colour in this figure legend, the reader is referred to the Web version of this article.)

Our findings demonstrated that a dose threshold of 60 J/cm^2^ was critical for promoting epidermal thickening and enhancing melanin secretion. As illustrated in Fig. 3A, exposure to blue light has been shown to induce KCs proliferation while reducing MCs viability. KCs are capable of secreting ET-1, which stimulates MCs to produce abundant melanin. The pathological alterations to the skin induced by blue light involve defense mechanisms, including epidermal thickening and increased melanogenesis.

### 3.4 PDA NPs and CINPs inhibit blue light-evoked severe oxidative stress and melanin production on epidermal cells

As depicted in Fig. 4A, PDA NPs and CINPs decrease blue light-induced ROS production, tyrosinase expression, and melanin concentration at the cellular level. Cytotoxicity assays revealed that PDA NPs and CINPs exhibited minimal toxicity towards KCs, even though their concentrations are up to 200 μg/mL (Fig. 4B), indicating their good biocompatibility and non-toxic nature. Sunscreens often generate deleterious ROS upon UV exposure, posing health risks. Simultaneously, blue light can also induce ROS production, leading to the oxidation of melanin precursors and stimulating melanin synthesis. We further assessed ROS levels to determine whether these nanoparticles could mitigate ROS generation. As illustrated in Fig. 4C and D, blue light irradiation was observed to enhance ROS production, while the incorporation of PDA NPs and CINPs was found to markedly diminish ROS levels in both KCs and MCs. PDA NPs and CINPs demonstrate sustained antioxidant capacity under repeated blue light exposure. As illustrated in Fig. S3, a blue light irradiation dose of 20 J/cm^2^ was administered on days 1, 3, and 5, and the intracellular ROS levels were measured at various time points. The results indicated that the ROS levels in the BL + PDA and BL + CINP groups were significantly lower compared to those in the BL group on days 1, 3, and 5. Moreover, on day 7, a reduced ROS level was maintained even in the absence of further irradiation. This effect was further validated in a co-culture system of KCs and MCs. After incubating KCs with nanoparticles for 6 h, the cells were irradiated with blue light and subsequently co-cultured with MCs for 24–48 h. Following irradiation, there was an elevation in tyrosinase expression in MCs. However, pre-treatment with PDA NPs and CINPs resulted in a significant reduction in tyrosinase expression. (Fig. 4E). Moreover, after 48 h of co-culture, the melanin content in MCs exhibited a comparable pattern, with the nanoparticle treatment leading to decreased melanin production (Fig. 4F). Subsequently, further investigations were conducted into the uptake and metabolic pathways of the nanoparticles. Electron microscopy confirmed the presence of nanoparticles within the cytoplasm of KC cells, where they were encapsulated in vesicles but did not enter the nucleus, forming protective melanin-like vesicles (Fig. 4G). Nanoparticles are eventually discharged after being encapsulated by lysosomes, resembling the natural processes of melanin synthesis and transport. We also analyzed the residual effect of nanoparticles. As shown in Fig. S4, at the cellular level, the retention of NP within KC was dynamically monitored by transmission electron microscopy on consecutive days 0, 1, 3, 5, 7, and 14. The melanin content within keratinocytes was detected by the NaOH method to quantify the residue of nanoparticles. Nanoparticles were gradually expelled from the cells from 5 to 14 days, with only a small amount remaining at 7 days and almost all being expelled at 14 days. The immunofluorescence results showed that PDA NPs and CINPs accumulated around the nuclear membrane, forming cap-like structures at the poles of the nucleus following blue light irradiation (Fig. 4H), consistent with previous reports [43]. Under blue light exposure, lysosomes were activated, playing a crucial role in the metabolism of the nanoparticles. These findings suggest a viable strategy for developing both artificial and natural melanin supplements, providing an alternative for endogenous melanin synthesis.

### 3.5 PDA NPs and CINPs inhibit blue light-induced pigmentation via the FZD2-TYR-melanin pathway

**Fig. 5:**
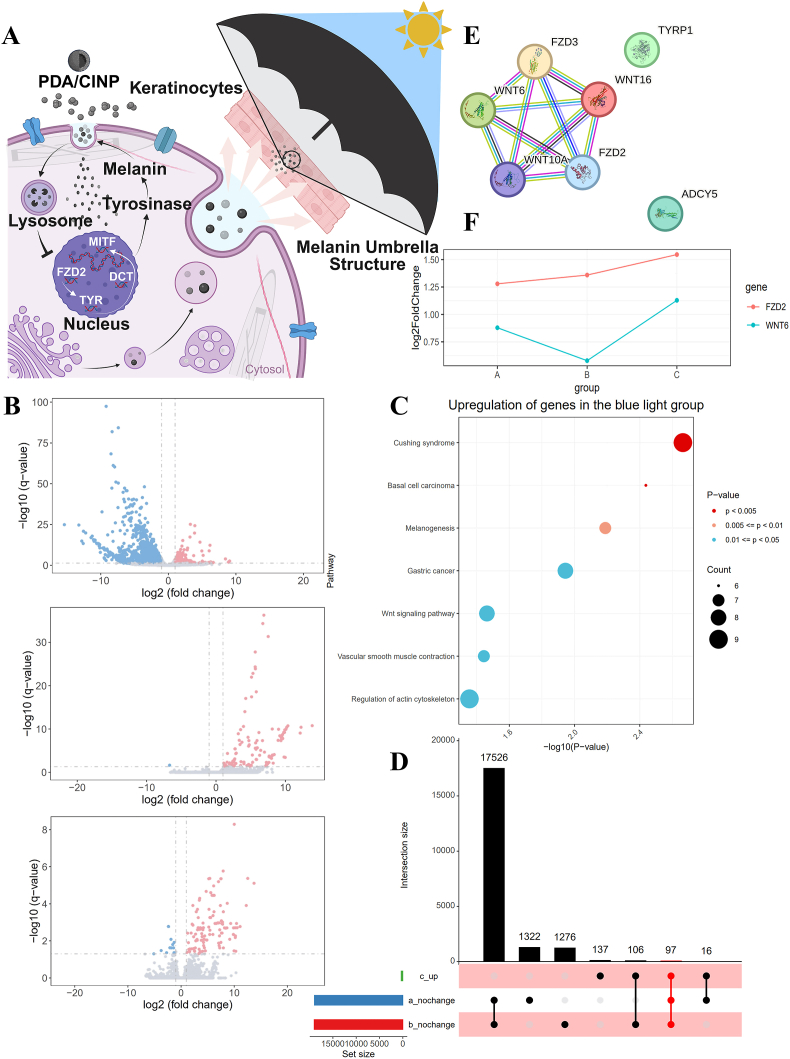
**Transcriptome sequencing reveals that nanoparticles inhibited blue light-induced hyperpigmentation either via FZD2 or WNT6.** (A) Scheme of the structure of the physical umbrella and the mechanism of the FZD2-dominated molecular pathway of nanoparticles. (B) Volcano plots of differential gene analysis of the blue light group, the blue light + PDA NPs group, and the blue light + CINPs group versus the control group (C) Differential gene KEGG analysis plots of the upregulated genes related to melanogenesis. (D) Gene crossover plot with genes upregulated after blue light intervention and not upregulated after nanoparticle intervention. a for BL + PDA; b for BL + CINP; c for BL. (E) PPI network plot analyzing melanogenesis-related Hub genes. (F) Comparative fold change analysis plot with nanoparticles reducing gene expression in the blue light treated group. A for BL + PDA; B for BL + CINP; C for BL. (For interpretation of the references to colour in this figure legend, the reader is referred to the Web version of this article.)

As shown in Fig. 5A, nanoparticles penetrate the epidermal keratinocyte cells to provide a protective umbrella for the skin, and the nanoparticles form melanin vesicle structures, which play the same protective role as normal melanin and participate in metabolic processes with the help of lysosomes. To further investigate whether PDA NPs and CINPs exert their effects on anti-melanin deposition through specific mechanisms, the differential analysis revealed a general downregulation of genes in the blue light-treated group compared to the control group (Fig. 5B). KEGG analysis indicated that the majority of the upregulated genes in the blue light-intervened group were associated with melanin synthesis (Fig. 5C). To identify protective genes linked to nanoparticle treatment, we selected 97 candidate genes that were upregulated in the blue light-intervened group while were not significantly elevated in the nanoparticle-intervened group (Fig. 5D). Protein-protein interaction (PPI) network analysis of genes related to melanin synthesis identified FZD2 and WNT6 as key regulatory genes (Fig. 5E). Fold-change analysis (Fig. 5F) indicated that these genes were less upregulated in the nanoparticle-intervened group than in the blue light-intervened group, suggesting that nanoparticles mitigate the impacts of blue light on these genes. This implies that the inhibition of melanin production by nanoparticles may involve FZD2 and WNT6.

**Fig. 6:**
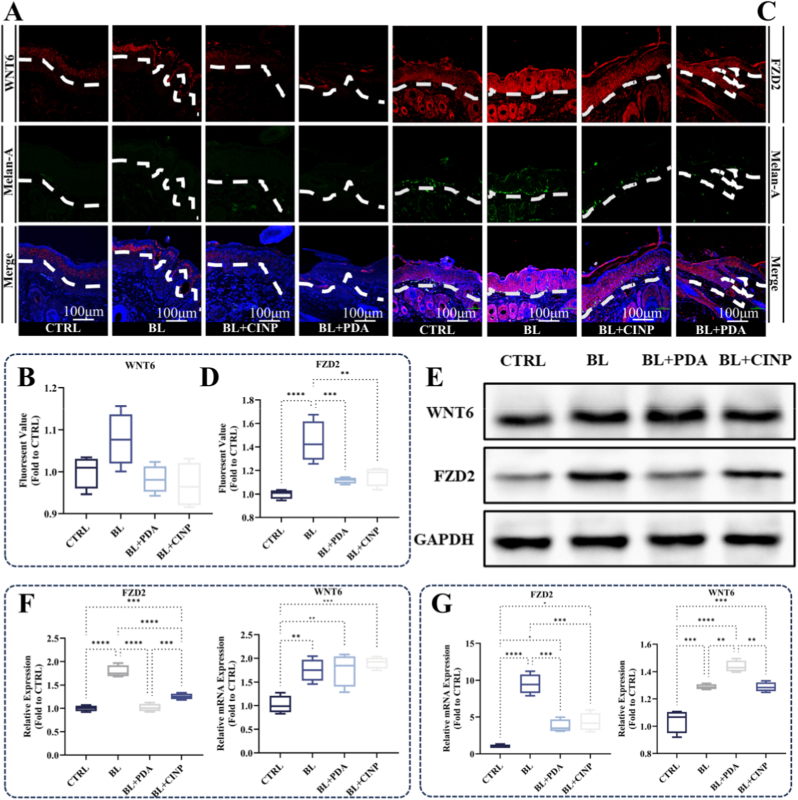
**PDA and CINPs inhibit blue light-induced FZD2 upregulation in guinea pig skin.** (A, C) Immunofluorescence staining sections of the skin of modeled animals, FZD2/WNT6 were labeled in red and Melan-A in green. The results demonstrated that FZD2 was significantly activated after blue light intervention, while no significant change was observed in FZD2 expression after nanoparticle intervention. There is no significance found in WNT6, scale bar = 100 μm. (B, D) Quantification of immunofluorescence. (E) WB experiment of animal samples, FZD2 was strongly activated after blue light intervention, while its expression was reduced after nanoparticle intervention. (F) Quantitative analysis of WB experiments of animal samples. (G) Quantitative analysis of RT-qPCR experiments of FZD2 and WNT6 in animal samples. All data are demonstrated as SD ± mean. ∗*P* < 0.05, ∗∗*P* < 0.01 and ∗∗∗*P* < 0.001. (For interpretation of the references to colour in this figure legend, the reader is referred to the Web version of this article.)

**Fig. 7:**
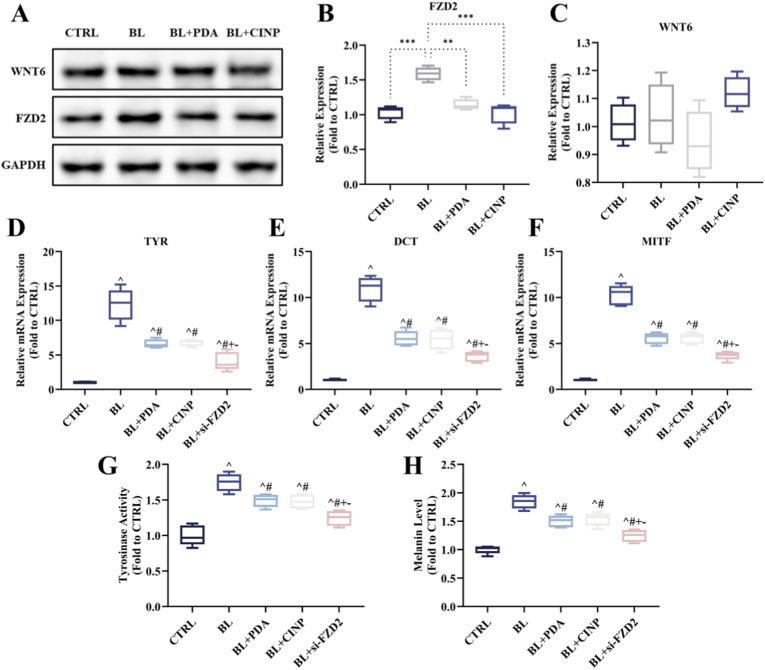
**PDA NPs and CINPs inhibit blue light-induced melanogenesis via FZD2 in melanocytes.** (A) WB experiment of melanocytes, FZD2 was strongly activated after blue light intervention in MCs, and its expression was reduced in the nanoparticle-treated group. There is no significance found in WNT6. (B, C) Quantitative analysis of WB experiment of melanocytes. (D–F) q-PCR experiment to verify TYR, DCT, and MITF expression after nanoparticle treatment and transfection of si-FZD2. (G, H) Tyrosinase activity assay, melanin detection NaOH measuring method, and nanoparticle treatment reduced tyrosinase activity and melanin production by silencing FZD2. All data represent the mean ± SD. ∗*P* < 0.05, ∗∗*P* < 0.01 and ∗∗∗*P* < 0.001, or ^^^*P* < 0.05 vs. CTRL, ^#^*P* < 0.05 vs. BL, ^+^*P* < 0.05 vs. BL + PDA, ^−^*P* < 0.05 vs. BL + CINP. (For interpretation of the references to colour in this figure legend, the reader is referred to the Web version of this article.)

In vitro experiments, including immunofluorescence (Fig. 6A–D), Western blotting (Fig. 6E and F), and RT-qPCR (Fig. 6G) assays, revealed that FZD2 expression was upregulated in epithelial tissues following blue light treatment. However, PDA NPs and CINPs suppressed the impact of blue light on FZD2 expression. Conversely, the expression of WNT6 was not significantly affected by blue light or nanoparticles. These findings were further validated in vitro in melanocytes and were consistent with the aforementioned results (Fig. 7A–C), indicating the accommodation of nanoparticles on FZD2 in melanocytes. Furthermore, following treatment with PDA NPs and CINPs, melanocytes exhibited a significant decrease in tyrosinase (TYR) activity, melanin levels, and the expression level of melanin-related genes, including TYR, dopachrome tautomerase (DCT), and microphthalmia-associated transcription factor (MITF), as illustrated in Fig. 7D–H. This was consistent with the silencing of FZD2. Collectively, these results showed that PDA NPs and CINPs could impede melanin deposition in melanocytes through the FZD2-TYR-melanin pathway. To further verify, FZD2 overexpressing human primary melanocytes (MCs) treated with PDA NPs or CINPs as shown in Fig. S5, q-PCR analysis was conducted. The results demonstrated that, compared to the FZD2 overexpression group (OE group), the expression levels of key transcription factors involved in melanin synthesis—TYR, DCT, and MITF—were significantly downregulated in both the OE + PDA group and the OE + CINP group. Additionally, measurements of tyrosinase activity and melanin content confirmed that PDA NP and CINP treatments effectively reversed the OE-induced increase in tyrosinase activity and significantly inhibited melanin production.

## 4 Discussion

Blue light holds a remarkable ability to penetrate the skin, reaching the dermis layer and potentially causing damage that includes aging, inflammation, dehydration, dryness, and chronic pigmentation [[43], [44], [45], [46]]. Epidermal melanin, as a pigment generated from melanocytes in the epidermis, acts as a pivotal component in the cutaneous self-photoprotection mechanism, helping to protect against the damaging effects of solar radiation. Acting as a natural sunscreen, melanin absorbs and dissipates solar radiation while also removing free radicals to minimize any potential harm from ultraviolet and visible light [[47], [48], [49]]. In this study, we characterized the morphology, structure, and zeta potential of synthetic PDA NPs and natural CINPs. These bioinspired nanoparticles exhibit structural similarities to endogenous melanin found in human skin. UV–Vis absorption spectroscopy confirmed that both types of nanoparticles show substantial absorbance in the blue light range, indicating a high potential for blue light attenuation. By employing PDA NPs and CINPs melanin analogs, we observed that their morphology and functions are highly analogous. Both nanoparticles displayed a stable UV–Vis absorbance profile and structural robustness, which were fundamental qualities required for effective blue light protection [38,39]. Moreover, we discovered that PDA and CINPs, as exogenous melanin, could not only mimic the endogenous melanin but also block melanogenesis via the FZD2-TYR pathway. These findings underscore the promising role of PDA and CINPs as viable agents in blue light protection.

Keratinocytes constitute over 90 % of cells of the epidermis and function as a key component in photoprotection. Relevant studies have demonstrated that blue light could influence the proliferation and differentiation of keratinocytes. For instance, low-intensity blue light may stimulate the growth and proliferation of keratinocytes, while high-intensity blue light can trigger a cellular stress response and inhibit the normal keratinization process [50]. In this study, we observed that the critical threshold for promoting epidermal thickening was 60 J/cm^2^ of low-dose blue light exposure. Melanocytes, situated mainly in the stratum basale of the epidermis of the skin, are another important target affected by blue light and produce a substantial amount of melanin when exposed to blue light. Furthermore, our results revealed that synthesized PDA NPs and natural CINPs as exogenous melanin not only attenuated blue light-induced keratinocyte-mediated epithelial thickening but also significantly reduced melanocyte-mediated melanin deposition in vivo.

ROS can cause oxidative damage to cell membranes, DNA, and proteins, leading to cellular dysfunction and death, while also activating and exacerbating inflammatory responses, ultimately accelerating skin aging. Our studies indicated that exposure to blue light stimulated keratinocytes and melanocytes to produce large amounts of ROS [51,52]. Simultaneously, related studies have shown that traditional sunscreens degrade under sunlight, releasing ROS that oxidize melanin precursors and promote melanogenesis [53]. The PDA has abundant functional groups, such as -NH and −OH, along with a quinone structure in adjacent positions, which enable it to trap and scavenge free radicals, thereby exhibiting antioxidant properties. Additionally, CINPs also contain -NH and -OH groups that neutralize free radicals. In this study, we successfully obtained the monodisperse spherical PDA NPs and CINPs, demonstrating that the incorporation of PDA NPs and CINPs dramatically scavenged ROS in keratinocytes and melanocytes and can show sustained clearance under repeated blue light irradiation. Furthermore, these nanoparticles suppressed tyrosinase expression and melanin formation. These results are in line with those of prior studies, suggesting that PDA NPs and CINPs have strong antioxidant potential against blue light exposure.

Biocompatibility is an essential property for candidates intended for blue light protection. Our results showed that both PDA NP and CINCP were not cytotoxic to keratinocytes at a high concentration of 200 μg/mL, whereas the conventional sunscreen Fe_2_O_3_/ZnO exhibited dose-dependent toxicity. The PDA NPs were found to be swallowed by keratinocytes in the absence of cytotoxicity and aggregated around the nucleus (called a perinuclear cap) in a previous study, protecting the nucleus from UV radiation, similar to natural melanin vesicles. In our study, we provide additional evidence that when exposed to blue light, both PDA NPs and CINPs were endocytosed into the cells and congregated around the nuclear membrane, akin to the natural melanosome 'caps' that protect the nucleus. Subsequently, PDA NPs and CINPs could be expelled from keratinocytes via exocytosis or degraded through lysosome activation. Thus, PDA NPs and CINPs can contribute to blue light protection both extracellularly and intracellularly. The cytotoxicity of PDA NPs and CINPs to keratinocyte viability, along with their intracellular degradability, suggests potential applications in sunscreens.

Blue light exposure can lead to more persistent pigmentation than UV light. Consequently, an in-depth exploration of the specific mechanisms by which nanoparticles mitigate blue light-mediated melanosis is essential. As we know, FZD2 can activate the classical WNT/β-catenin signaling pathway, which is correlated closely with melanin synthesis [54,55]. Through transcriptome analysis and subsequent validation, we identified FZD2 as a potential hub gene in the blue light-mediated pigmentation response, and nanoparticle treatment could remarkably inhibit key downstream melanogenesis factors (e.g., TRY, DCT, and MITF) via FZD2, which may partially shed light on the mechanism of the anti-pigmentation effect of exogenous melanin.

Although our results indicate that PDA NPs and CINPs are highly effective photoprotective agents for blue light, it is vital to acknowledge the limitations of our study. It is important to note that this experiment only confirmed that PDA and CINPs can inhibit melanin formation through FZD2. Further investigation into the regulatory differences of the molecular pathways between synthetic PDA NPs and natural CINPs to blue light resistance is lacking. Our previous study found that 5 % WT PDA exhibited a favorable effect on UV resistance and this experiment also confirmed that 5 % WT PDA NPs and CINPs had a superior efficacy in blue light resistance [26].

Overall, our results deciphered that bioinspired PDA NPs and CINPs, as promising exogenous melanin supplements, possess robust photoprotection ability against blue light. This photoprotection may contribute to their effects on antioxidation and the reduction of pigmentation. The underlying mediators and mechanisms by which PDA NPs and CINPs reduce melanin formation involve the inhibition of the FZD2-TYR-melanin pathway.

## 5 Conclusion

Taken together, PDA NPs and CINPs, as novel bioinspired materials designed to mimic natural melanin, can effectively withstand blue light-induced melanin deposition. Our results demonstrated that both PDA NPs and CINPs exhibited high stability, excellent biocompatibility with keratinocytes and melanocytes, and efficient absorption and scattering of blue light. Moreover, they had antioxidant properties that reduce ROS generation in keratinocytes and melanocytes following blue light exposure, thereby mitigating oxidative stress during melanin synthesis. *In vitro* experiments confirmed that sunscreens containing PDA NPs and CINPs performed superior in reducing blue light-induced melanin deposition and epidermal hyperplasia. Otherwise, we identified the FZD2 as a potential target gene for lowering melanin synthesis, and PDA NPs and CINPs were able to downregulate FZD2 expression, which in turn reduced the expression of TRY, DCT, and MITF, effectively suppressing melanin formation. Given their safety, environmental friendliness, and efficacy, PDA NPs and CINPs as exogenous melanin supplements could be harnessed for cosmetic and dermatological applications, thereby opening up new avenues for skin protection formulations aiming specifically at blue light-induced pigmentation.

## CRediT authorship contribution statement

**Xiaoqi Chen:** Writing – original draft, Visualization, Methodology, Investigation, Formal analysis, Data curation. **Tong Wu:** Writing – review & editing, Visualization, Methodology, Investigation, Formal analysis, Data curation. **Zijun Chen:** Writing – review & editing, Visualization, Methodology, Investigation, Formal analysis, Data curation. **Jia Zhang:** Methodology, Investigation, Formal analysis. **Yuqi Zhou:** Methodology, Investigation, Formal analysis. **Qi Wang:** Methodology, Investigation, Formal analysis. **Bo Wang:** Methodology, Investigation, Formal analysis. **Zeqian Wang:** Methodology, Investigation, Formal analysis. **Xiaodong Jin:** Methodology, Investigation, Formal analysis. **Shishi Xiong:** Methodology, Investigation, Formal analysis. **Tong Zhang:** Methodology, Investigation, Formal analysis. **Shanshan Gao:** Methodology, Investigation, Formal analysis. **Jingjing Ma:** Methodology, Investigation, Formal analysis. **Ziwei Deng:** Writing – review & editing, Supervision, Conceptualization. **Xutao Chen:** Writing – review & editing, Supervision, Project administration, Conceptualization. **Chunying Li:** Writing – review & editing, Supervision, Project administration, Methodology, Investigation, Formal analysis, Data curation, Conceptualization. **Zhe Jian:** Writing – review & editing, Supervision, Project administration, Methodology, Investigation, Funding acquisition, Formal analysis, Data curation, Conceptualization.

## Funding

This work was supported by the 10.13039/501100001809National Natural Science Foundation of China (No. 82173414 and 82373473 granted to Zhe Jian); Key projects of the Shaanxi Natural Science Basic Research Program (2023-JC-ZD-44 granted to Zhe Jian); Flying Plan of Ling yun Project of 10.13039/501100007547Fourth Military Medical University (axjhjz-102 granted to Zhe Jian); and The Postdoctoral Fellowship Program of 10.13039/501100002858China Postdoctoral Science Foundation (No. GZC20233581 granted to Xutao Chen).

## Declaration of competing interest

The authors declare that they have no known competing financial interests or personal relationships that could have appeared to influence the work reported in this paper.

## Data Availability

Data will be made available on request.
