# Supplementary material for: Bioinspired nanoparticles prevent blue-light-induced skin hyperpigmentation via FZD2-TYR-melanin pathway
Source: Mater Today Bio. 2025 Sep 4;35:102288. doi: 10.1016/j.mtbio.2025.102288 (PMC12449806; doi:10.1016/j.mtbio.2025.102288)
Supplement: Multimedia component 1 [file mmc1.docx]

**Supporting Information**

**Bioinspired Nanoparticles Prevent** **blue-light-induced Skin Hyperpigmentation via FZD2-TYR-melanin pathway**

*Xiaoqi Chen^1*^, Tong Wu^1*^, Zijun Chen^2*^, Jia Zhang^1^, Yuqi Zhou^1^, Qi Wang^1^, Bo Wang^1^, Zeqian Wang^1^, Xiaodong Jin^1^, Shishi Xiong^1^, Tong Zhang^1^, Shanshan Gao^1^**, Jingjing Ma^1^,* Ziwei Deng*^4#^, Xutao Chen**^3#^, Chunying Li^1#^ and Zhe Jian^1#^*

^1^ Department of Dermatology, Xijing Hospital, Fourth Military Medical University, Xi’an, Shaanxi 710032, P. R. China.

^2^ Faculty of Dentistry, The University of Hong Kong, Hong Kong 999077, P. R. China

^3^ Department of Immunology, School of Basic Medicine, Fourth Medical University, Xi'an, Shaanxi 710032, P. R. China

^4^ Key Laboratory of Applied Surface and Colloid Chemistry, Ministry of Education，School of Materials Science and Engineering, Shaanxi Normal University, Xi'an 710119, P. R. China.

**Corresponding author:**

Zhe Jian (E-mail: xjzhejian@fmmu.edu.cn)

Chunying Li (E-mail: lichying@fmmu.edu.cn)

Xutao Chen (E-mail: [cxt1083583097@163.com](mailto:cxt1083583097@163.com))

Ziwei Deng (E-mail: zwdeng@snnu.edu.cn)

^*^ These authors contributed equally to this work

^#^ Corresponding Author

1. **Materials and Methods**

**1.1 Lentiviral infection**

The lentiviral vector pLV-Zs Green (2A) PURO-CMV overexpressing FZD2 was purchased from Zingke Biotechnology (Beijing, China). Human primary melanocytes were infected with lentivirus at an MOI of 10 and screened with puromycin at a concentration of 10 μg/mL followed by passaging culture and experiments.

**1.2 Animal Models**

Female brown guinea pigs (4-6 weeks old) were acclimatised to the environment for 1 week, and the dorsally shaved skin of the guinea pigs in the experimental group was divided into four sections corresponding to the four treatments: application of PDA NPs or CINPs containing 1 wt%, 2.5% wt%, 5% wt%, 10% wt%, and simultaneous irradiation of the four areas with blue light. The PDA NPs or CINPs were simply mixed with the emulsion, which was used as a nanomaterial carrier.

At the same time, the back of guinea pigs from another experimental group was divided into four regions corresponding to four treatments: application of PDA NPs containing 5 wt%; 5 wt% CINPs; 5% zinc oxide; 5% iron oxide, and the four regions were irradiated with blue light simultaneously. The PDA NPs, CINPs, zinc oxide, and iron oxide were simply mixed with the emulsion, which was used as a carrier for the nanomaterials. All animal sample experiments were repeated 4 times.

**1.3 PDA NPs and CINPs residual assay**

The melanin content of keratinocytes was measured by NaOH assay at different time points after incubating keratinocytes with 40 μg/ml PDA NPs or CINPs for 24 h. This assay was used to quantify the melanin residuals of PDA NPs and CINPs in keratinocytes. The intracellular residues of PDA NPs, CINPs at different time points were directly observed by transmission electron microscopy (80.0kV Hitachi 7800 TEM system).

**1.4 Iron oxide and zinc oxide**

Red Fe_2_O_3_ (~25g) and ZnO (~500g)^1,2^ were purchased from Merck (# 310050,# 1088490500) and were tested for protection against blue light by topically applying them to the back skin of guinea pigs after simply mixing them with an emulsion.


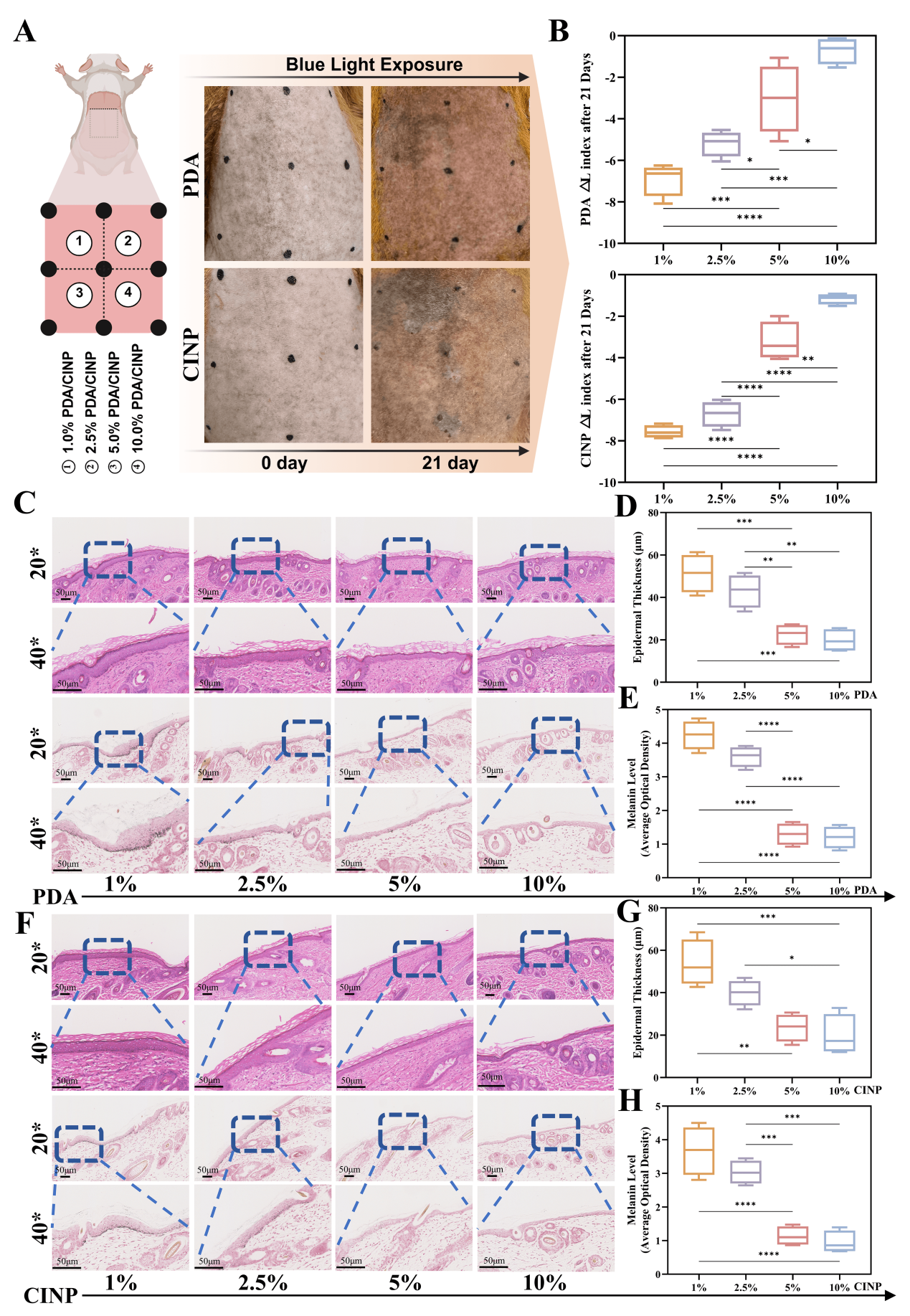


**Figure S1.** The role of different concentrations of PDA NPs and CINP NPs in attenuating blue light-induced hyperpigmentation. (A) Schematic showing the topical application of PDA NPs/CINP on the dorsal skin of brown guinea pigs. Different concentrations of 1%wt, 2.5%wt, 5%wt and 10%wt are shown on the upper left, upper right, lower left and lower right, respectively. Representative images of hyperpigmentation on the dorsal side of brown guinea pigs before and after treatment. (B) Changes in skin pigmentation were assessed spectrophotometrically and ΔL values were quantified for different areas. (C) Images of H&E and Fontana-Masson staining of skin after blue light irradiation with different concentrations of PDA. (D) Quantitative statistics of H&E epidermal thickness of PDA. (E) Quantitative statistics of Fontana-Masson melanin levels of PDA. (F) Images of H&E and Fontana-Masson staining of skin after blue light irradiation with different concentrations of CINP. (G) Quantitative statistics of H&E epidermal thickness of CINP. (H) Quantitative statistics of Fontana-Masson melanin levels of CINP. All data are expressed as standard deviation ± mean. **P* < 0.05, ***P* < 0.01, ****P* < 0.001, and *****P* < 0.0001.


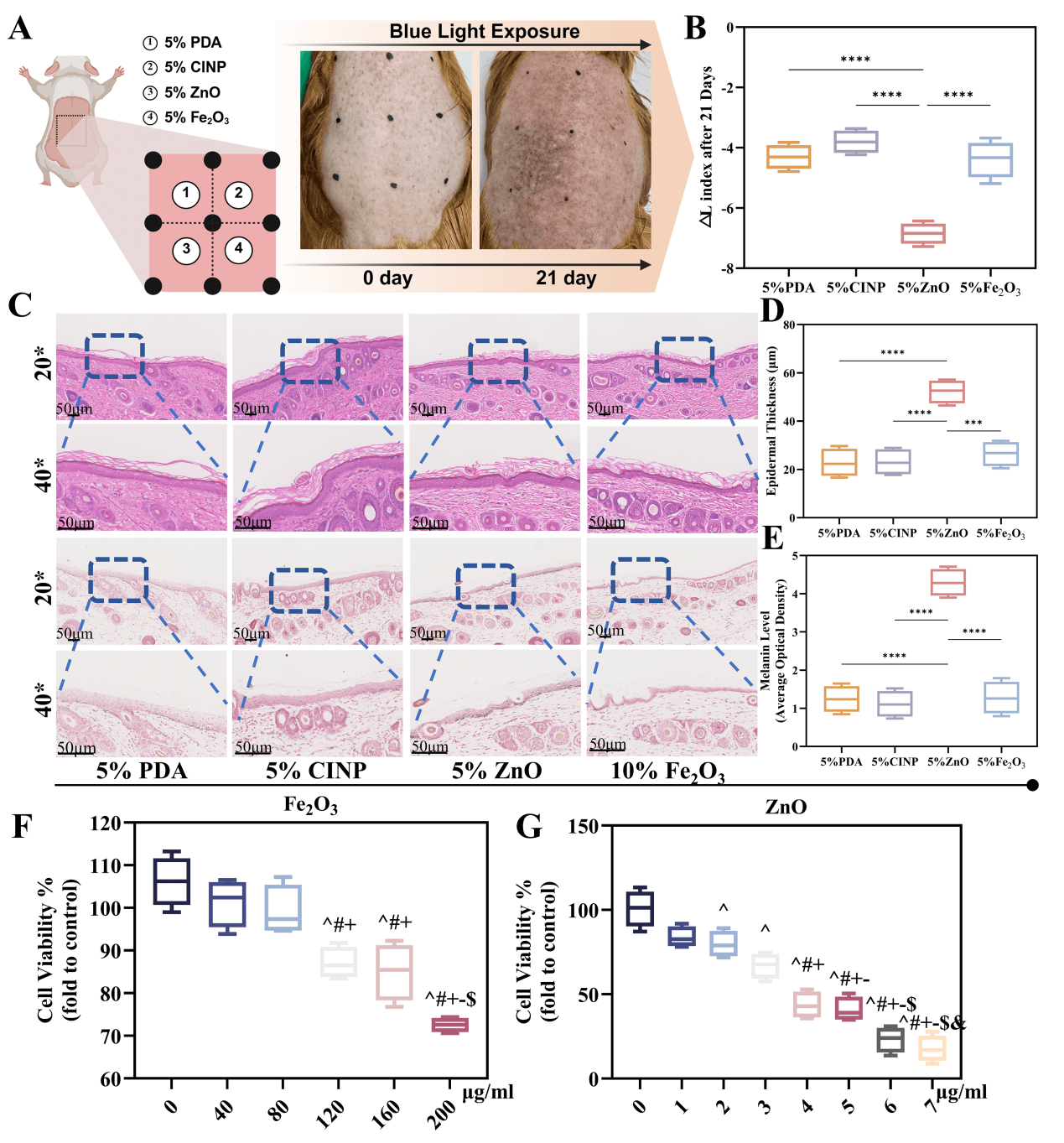


**Figure S2.** Role of PDA NPs, CINPs, zinc oxide and iron oxide on blue light-induced hyperpigmentation. (A) Schematic showing topical application of nanomelanin on the dorsal skin of a brown guinea pig. Top left, top right, bottom left, and bottom right are 5%wt PDA NPs, 5%wt CINP NPs, 5%wt zinc oxide, and 5%wt iron oxide, respectively. Representative images of dorsal hyperpigmentation after blue light irradiation of different nanomaterials. (B) Changes in skin pigmentation were assessed spectrophotometrically and ΔL values were quantified for different areas. (C) H&E and Fontana-Masson staining images of the skin. (D) Quantification of epidermal thickness in HE. (E) Quantitative statistics of melanin levels in FM. (F-G) The CCK8 method was used to evaluate the effects of different doses of iron oxide and zinc oxide on the viability of human primary keratinocytes (KCs). All data are expressed as standard deviation ± mean. ****P* < 0.001, and *****P* < 0.0001 or ^^^*P* < 0.05 vs. 0 μg/ml, ^#^*P* < 0.05 vs. 40 μg/ml, ^+^*P* < 0.05 vs. 80 μg/ml, ^-^*P* < 0.05 vs. 120 μg/ml, ^$^*P* < 0.05 vs. 160 μg/ml or ^^^*P* < 0.05 vs. 0 μg/ml, ^#^*P* < 0.05 vs. 1 μg/ml, ^+^*P* < 0.05 vs. 2 μg/ml, ^-^*P* < 0.05 vs. 3 μg/ml, ^$^*P* < 0.05 vs. 4 μg/ml, ^&^*P* < 0.05 vs. 5 μg/ml.


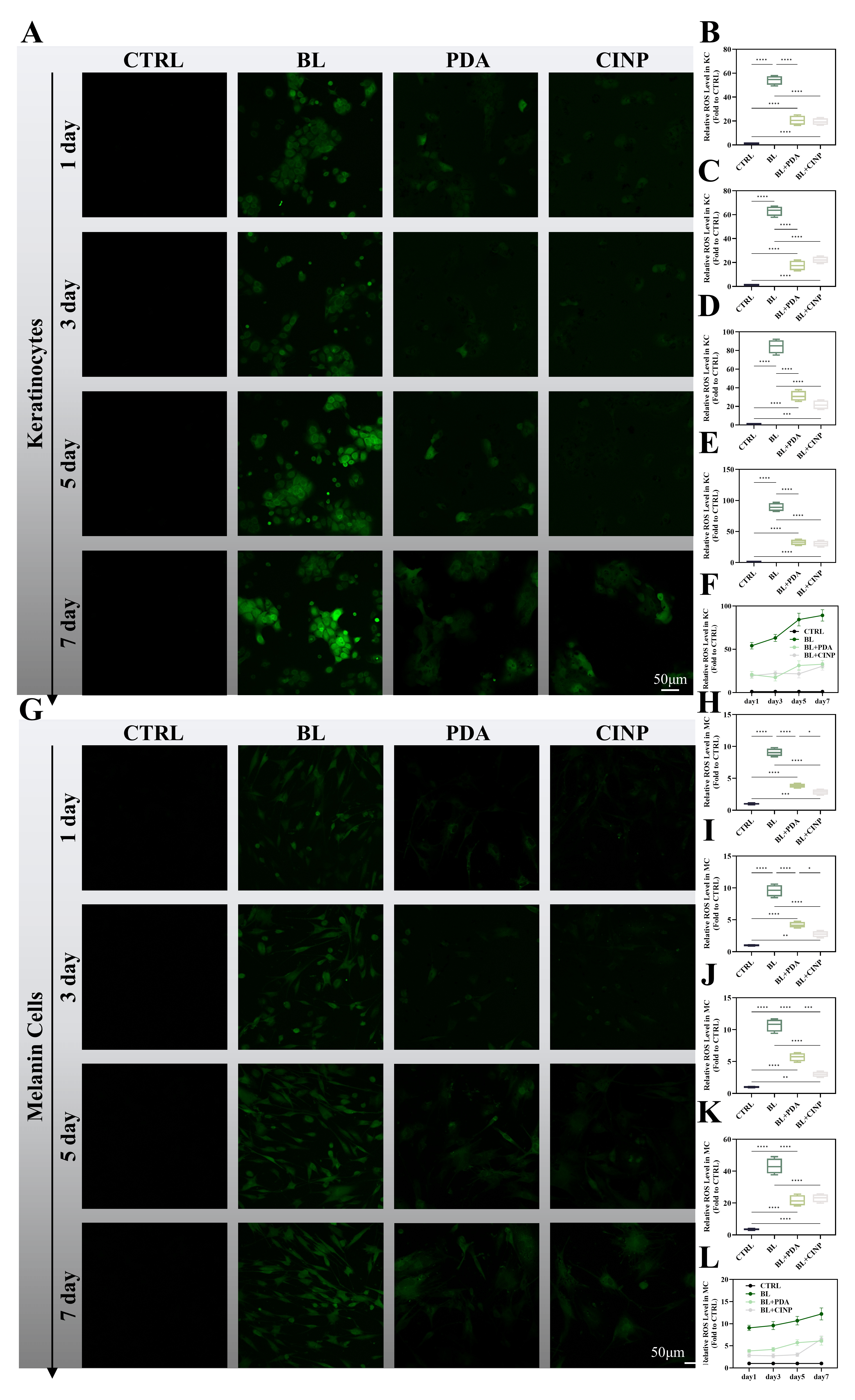


**Figure S3.** Reduction of blue light-induced ROS production by PDA NPs and CINPs under multiple blue light irradiation. (A) KCs were treated with 40 μg/mL PDA NPs or CINCs. ROS levels were assessed through blue light irradiation on days 1, 3, and 5. On day 7, in the absence of irradiation, the potential rebound and increase in ROS levels were monitored. Representative fluorescence images were obtained using confocal microscopy. (G) MCs were treated with 40 μg/mL PDA NPs or CINCs. ROS levels were assessed through blue light irradiation on days 1, 3, and 5. On day 7, in the absence of irradiation, the potential rebound and increase in ROS levels were monitored. Representative fluorescence images were obtained using confocal microscopy. (B-F) DCFH-DA (green) was used to quantify ROS levels, and fluorescence intensities in KC cells at different time points were counted and statistically analysed. (H-L) The ROS fluorescence intensity of MC cells at different time points was statistically analyzed. scale bar = 50 μm. All data are expressed as standard deviation ± mean. **P* < 0.05, ***P* < 0.01, ****P* < 0.001, and *****P* < 0.0001.


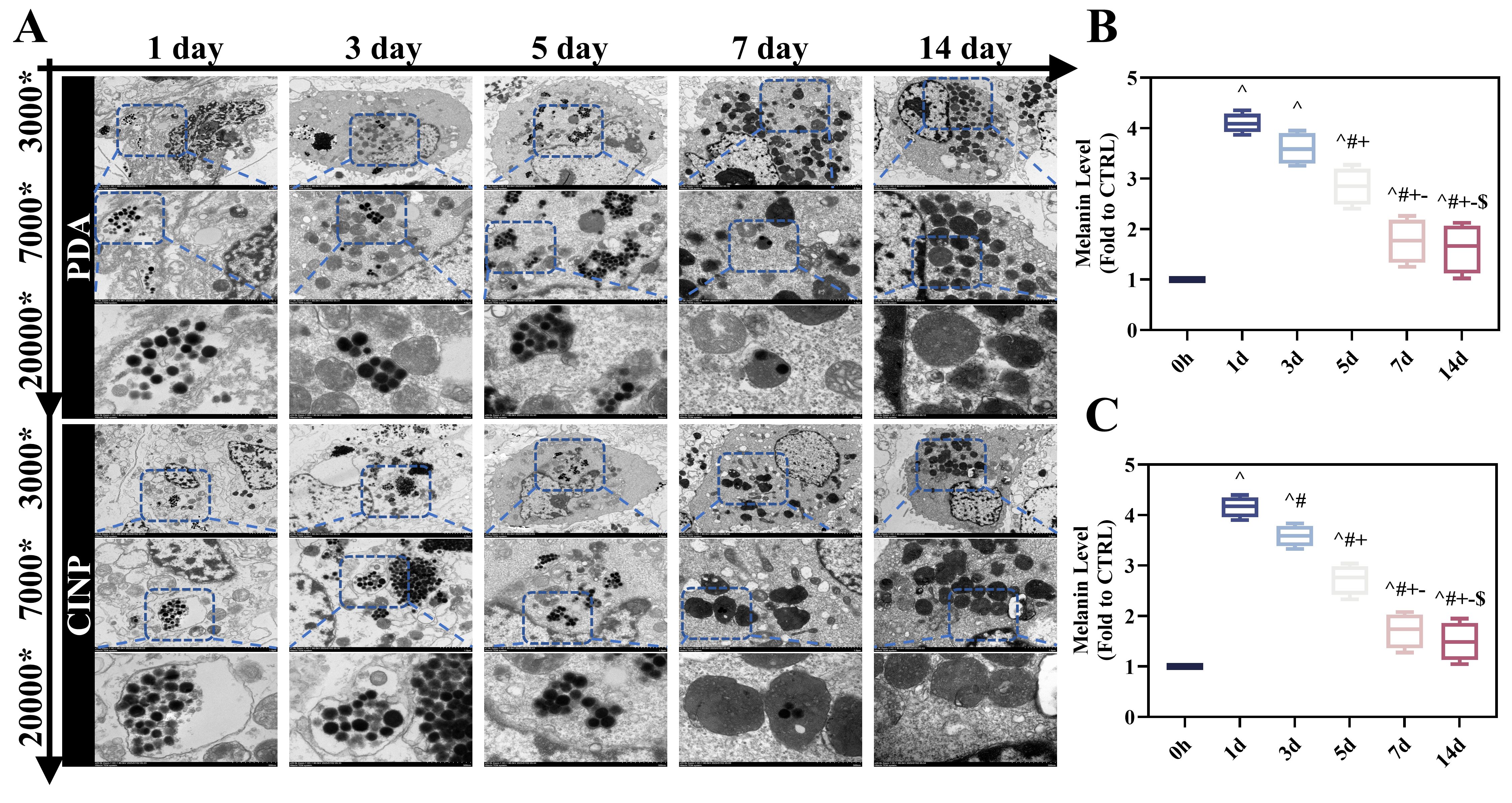


**Figure S4.** Residues of PDA NPs and CINPs in keratinocytes at different time points. (A) Cellular electron microscopy was performed to observe the metabolism of melanin nanoparticles (PDA NPs and CINPs) in keratinocytes at different time points, which were continuously cytophagocytosed into keratinocyte cytoplasm during 1-3d time, and the cells produced a large number of lysosomes and wrapped melanin nanoparticles during 3-7d time. Particles are gradually discharged out of the cell, and a large number of lysosomal particles can be observed at 7 days and 14 days under 20,000x magnification. It is shaped as a black sphere encasing melanin nanoparticles. (B) Residues of PDA NPs, NaOH method to detect melanin content in keratin-forming cells at different time points. 0 h is for comparison without adding PDA NPs. (C) Residual amount of CINPs, NaOH method to detect melanin content in keratin-forming cells at different time points. 0 h is for comparison without adding CINPs. The melanin content in keratinocytes was the highest at 1 day, gradually decreasing with the increase of time, and almost completely metabolised within 7-14d. All data are expressed as standard deviation ± mean. ^*P* < 0.05 vs. 0h, ^#^*P* < 0.05 vs. 1d, ^+^*P* < 0.05 vs. 3d, ^-^*P* < 0.05 vs. 5d, ^$^*P* < 0.05 vs. 7d.

**
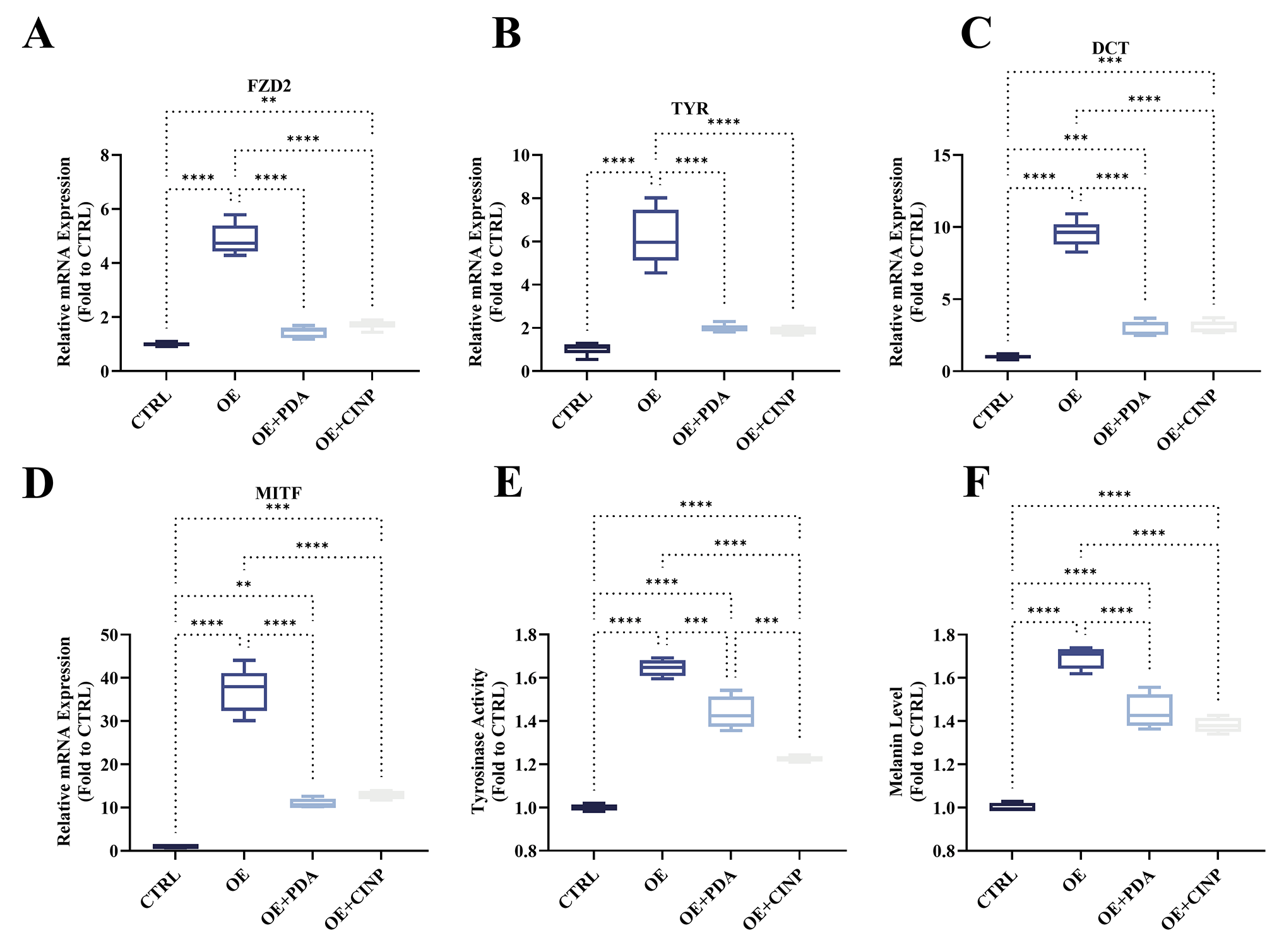
**

**Figure S5.** PDA NPs and CINPs inhibit melanogenesis-related transcription factors, tyrosinase and melanogenesis in melanocytes overexpressing FZD2. (A-D) q-PCR experiments were performed to verify the expression of FZD2, TYR, DCT and MITF in melanocytes overexpressing FZD2 and in melanocytes treated with PDA NPs and CINP NPs. (E-F) Tyrosinase activity assay, melanin detection NaOH measurement method to verify that PDA NPs, CINP NPs treatment inhibits tyrosinase activity and melanogenesis caused by overexpression. All data are expressed as standard deviation ± mean. ***P* < 0.01, ****P* < 0.001, and *****P* < 0.0001.

**References**

1 Bernstein, E. F., Sarkas, H. W. & Boland, P. Iron oxides in novel skin care formulations attenuate blue light for enhanced protection against skin damage. *Journal of Cosmetic Dermatology* **20**, 532-537, doi:10.1111/jocd.13803 (2020).

2 Schneider, S. L. & Lim, H. W. A review of inorganic UV filters zinc oxide and titanium dioxide. *Photodermatol Photoimmunol Photomed* **35**, 442-446, doi:10.1111/phpp.12439 (2019).
